# Cervical Ectopic Pregnancy After Frozen Embryo Transfer: Methotrexate Failure Managed With Uterine Artery Embolization and Ultrasound-Guided Curettage

**DOI:** 10.7759/cureus.98928

**Published:** 2025-12-10

**Authors:** Reem Saab, Megan Melnyk, Grace Ferguson, Justin Pilgrim

**Affiliations:** 1 Department of Obstetrics and Gynecology, Allegheny Health Network, Pittsburgh, USA

**Keywords:** assisted reproductive technology (art), cervical ectopic pregnancy, fertility preservation, frozen embryo transfer, methotrexate failure, uterine artery embolization (uae)

## Abstract

Cervical ectopic pregnancy is a rare but potentially life-threatening form of ectopic implantation that poses a significant risk for severe hemorrhage and loss of fertility. This case report describes a cervical ectopic pregnancy following frozen embryo transfer that failed multi-dose methotrexate therapy and was successfully managed with uterine artery embolization followed by ultrasound-guided dilation and curettage. A 37-year-old nulliparous woman with a history of bilateral salpingectomy and tubal factor infertility conceived after frozen embryo transfer. She received a multi-dose systemic methotrexate regimen consisting of four intramuscular doses over seven days with alternating oral leucovorin, followed by bilateral uterine artery embolization and immediate ultrasound-guided suction dilation and curettage.

On Day 0, the patient presented with vaginal bleeding and was diagnosed with a cervical ectopic pregnancy at approximately six weeks’ gestation. Initial β-hCG was >14,000 mIU/mL (reference value < 5 mIU/mL). She underwent multi-dose systemic methotrexate, but β-hCG declined only 13% by Day 2, 25% by Day 4, and then plateaued with <2% change between Day 4 and Day 6, consistent with methotrexate failure. She continued to experience persistent spotting. On Day 13, she underwent bilateral uterine artery embolization followed immediately by ultrasound-guided suction curettage. The procedure was uncomplicated, with minimal blood loss (5 mL) and no transfusion. Pathology confirmed gestational tissue, and she was discharged the same day with outpatient follow-up with her reproductive endocrinology and infertility physician.

Cervical ectopic pregnancy is a rare but potentially catastrophic condition, particularly in the context of assisted reproductive technology. In this case, high β-hCG levels and prior embryonic cardiac activity predicted poor response to methotrexate. Fertility-preserving surgical strategies, specifically uterine artery embolization followed by suction curettage, provided safe, definitive management and avoided hysterectomy. This case highlights the importance of early recognition of methotrexate failure, prompt multidisciplinary coordination, and timely transition to fertility-preserving surgical intervention to optimize outcomes in patients with cervical ectopic pregnancy.

## Introduction

The incidence of cervical ectopic pregnancies in all pregnancies that are not the result of assisted reproductive technology (ART) is less than 0.01%. Specifically, cervical ectopic pregnancies account for less than 1% of all ectopic pregnancies, and the overall incidence of ectopic pregnancy in the general population is approximately 1-2% of all pregnancies [1-4]. The incidence rate of cervical ectopic pregnancies after ART is 0.035% of all ongoing pregnancies, and cervical ectopic pregnancies represent 2.02% of all ectopic pregnancies following ART [5].

Without early recognition, surgical intervention is associated with catastrophic hemorrhage and high rates of hysterectomy [6,7]. Dilation and curettage (D&C) for cervical ectopic pregnancy (CEP) carries a particularly significant risk of severe hemorrhage because the cervix is richly vascular and has limited contractility compared with the uterine corpus [6-8]. Multiple case reports document life-threatening bleeding during or after D&C for cervical pregnancy, requiring emergency interventions including uterine artery embolization, balloon tamponade, cerclage, or even hysterectomy [2,6-10]. In a scoping review of 454 cases, 9% of patients ultimately required hysterectomy, and more than half needed multiple interventions to achieve resolution [7].

Methotrexate (MTX), administered systemically or locally, is often first-line therapy in hemodynamically stable patients. However, treatment success is strongly dependent on prognostic factors. Hung et al. identified four predictors of MTX failure: β-hCG ≥10,000 mIU/mL, gestational age ≥9 weeks, crown-rump length (CRL) >10 mm, and embryonic cardiac activity [11]. Reduced efficacy in viable cervical pregnancies has also been noted [12]. Although conservative medical approaches may succeed in early, favorable cases [13,14], failure necessitates surgical intervention.

Prophylactic measures can substantially reduce hemorrhagic complications when D&C is performed. Uterine artery embolization performed immediately before D&C has been shown to minimize bleeding and allow safe evacuation [15]. Local injection of diluted vasopressin into the cervix surrounding the gestational sac induces vasoconstriction and cervical smooth muscle contraction, facilitating removal with minimal blood loss [16]. Cervical cerclage placed before curettage, combined with intracervical carboprost infiltration and balloon tamponade, has successfully prevented severe hemorrhage [9].

Advances in ultrasound, particularly transvaginal ultrasound, have improved the diagnosis of CEP, with key features including a gestational sac located in the cervical canal, absence of intrauterine pregnancy, a negative “sliding sign,” and peritrophoblastic vascularity on Doppler [3,6].

Fertility-sparing surgical options incorporating hemostatic adjuncts have been increasingly reported. Fylstra demonstrated the safety of ultrasound-guided suction curettage followed by balloon tamponade in 13 consecutive cases [17], while Hu et al. described uterine artery embolization (UAE) followed by curettage as an effective strategy to minimize blood loss and preserve fertility [18-19]. Additional combined approaches, such as cerclage with pharmacologic agents and UAE with cervical tamponade, have also been reported to preserve fertility in CEP [9,18]. These approaches are especially relevant to patients conceiving with ART, in whom future fertility remains a priority.

We present a case of CEP following frozen embryo transfer (FET) that failed multi-dose MTX therapy and was successfully treated with UAE followed by ultrasound-guided suction curettage.

## Case presentation

The patient is a 37-year-old nulliparous woman with a three-year history of primary infertility despite regular unprotected intercourse. She initially presented to reproductive endocrinology and infertility (REI) specialists after failing to conceive with timed intercourse and ovulation tracking.

As part of her initial infertility workup, a transabdominal and transvaginal pelvic ultrasound performed in early 2023 demonstrated a normal-sized uterus (8.6 × 3.4 × 5.6 cm) with a 1.4-cm right corpus subserosal leiomyoma and normal-appearing ovaries with intact arterial and venous blood flow. A repeat pelvic ultrasound later that year showed a uterus measuring 7.0 × 3.8 × 6.6 cm with a partially exophytic heterogeneous mass arising from the posterior uterine body, measuring up to 1.7 cm and consistent with a fibroid. The cervix was unremarkable, the endometrial stripe measured 9 mm without abnormal vascularity, and both ovaries appeared normal with bilateral follicles; a 2.3-cm complex hypoechoic lesion in the left ovary was favored to represent a corpus luteum cyst. No free fluid was present.

Hysterosalpingography subsequently demonstrated an irregular filling defect of the endometrial cavity, a patent left fallopian tube, and right tubal occlusion, prompting further evaluation of the uterine cavity. She underwent operative hysteroscopy with D&C for menorrhagia and an endometrial abnormality. Intraoperatively, the cervix was noted to be stenotic and required dilation. Hysteroscopic inspection revealed a large polypoid area of tissue arising from the right lateral uterine wall that filled most of the cavity, along with several additional polypoid areas. These lesions were removed using polyp forceps and sharp curettage, with re-inspection confirming a normal-appearing cavity at the end of the procedure. Pathology demonstrated benign endometrial curettings consistent with an endometrial polyp.

At age 35, due to persistent pelvic pain and infertility, she underwent diagnostic and operative laparoscopy. Operative findings demonstrated extensive pelvic adhesive disease with distortion of normal adnexal anatomy. There were multiple endometriotic implants on the pelvic sidewalls and in the posterior cul-de-sac, with adhesions between the uterus and rectum, but no obvious bowel nodules. Both fallopian tubes appeared dilated and occluded; chromopertubation confirmed bilateral tubal occlusion, and a left hydrosalpinx was identified. A 2-3-cm right lateral fibroid was also noted. Surgical management included laparoscopic excision of visible endometriotic implants, adhesiolysis, and bilateral salpingectomy with removal of the hydrosalpinx. These findings confirmed tubal factor infertility in the setting of endometriosis, and in vitro fertilization (IVF) was recommended as her only option for conception.

Pre-IVF uterine cavity assessment also included a saline infusion sonohysterogram (SIS). Sagittal imaging showed a clear endometrial cavity with subtle “endometrial undermining”, a subtle irregularity characterized by a faint, shallow separation at the endometrial-myometrial junction without a discrete mass, polyp, or synechiae. Three-dimensional reconstruction confirmed a normal upper uterine segment and fundus; however, the lower uterine segment was suboptimally visualized. A repeat SIS was attempted, but catheter passage was unsuccessful due to cervical stenosis. Given her prior hysteroscopic treatment and the challenges with catheter passage, she ultimately proceeded with embryo transfer after operative hysteroscopy had confirmed a cavity suitable for implantation.

Her ovarian reserve testing was within the normal range for age (estradiol 39 pg/mL, FSH 6.1 mIU/mL, LH 4.4 mIU/mL, AMH 2.20 ng/mL), and she underwent controlled ovarian hyperstimulation using a gonadotropin antagonist protocol. Transvaginal oocyte retrieval yielded four mature oocytes. Intracytoplasmic sperm injection (ICSI) was used for fertilization, and several normally fertilized embryos (2PN) developed to the blastocyst stage. Preimplantation genetic testing for aneuploidy (PGT-A) identified two euploid blastocysts suitable for transfer.

She then began a programmed FET cycle. Endometrial preparation was achieved with exogenous estradiol followed by intramuscular progesterone in oil for luteal support. On lining check, the endometrium was appropriately thickened, and laboratory markers confirmed readiness for transfer. Using a Cook Soft-Pass EchoTip catheter under transabdominal ultrasound guidance, a single day-5 euploid blastocyst was transferred to the uterine fundus. The transfer was technically challenging because of her history of cervical stenosis and prior cervical dilation, but was ultimately atraumatic. The tip of the catheter was placed into the uterus under ultrasound guidance. The inner catheter was removed and replaced with the catheter containing the embryo. The fluid containing the embryo was released at approximately 1.5 cm from the uterine fundus. The catheter was slowly removed. The embryologist inspected the catheter to confirm that there was no retained embryo.

Her post-transfer course was monitored with serial β-hCG testing, which demonstrated appropriate interval rises consistent with early pregnancy. A viability ultrasound was scheduled for 7 weeks of gestation.

**Figure 1:**
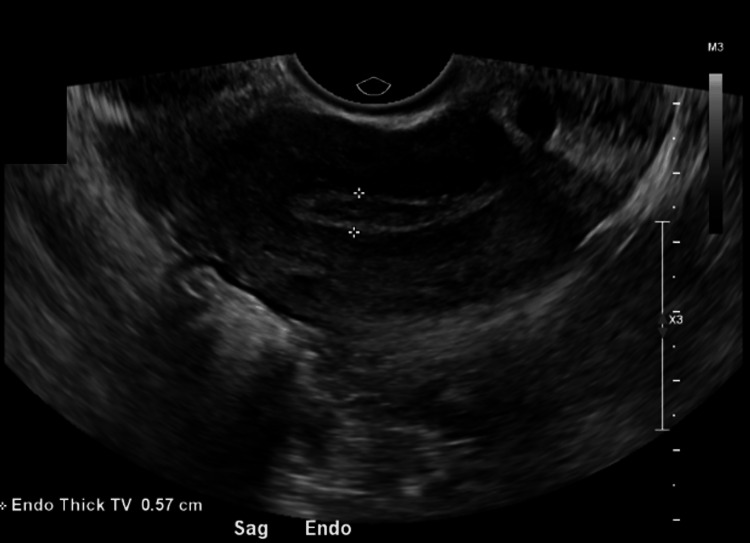
Transvaginal ultrasound on Day 0 showing absence of intrauterine gestation. Initial transvaginal ultrasound obtained on Day 0 demonstrates an empty uterine cavity with an endometrial thickness measuring 0.57 cm. The white arrowheads indicate the endometrial stripe, with no intrauterine gestational sac identified. Findings were concerning for an ectopic implantation, confirmed to be cervical in location.

**Figure 2:**
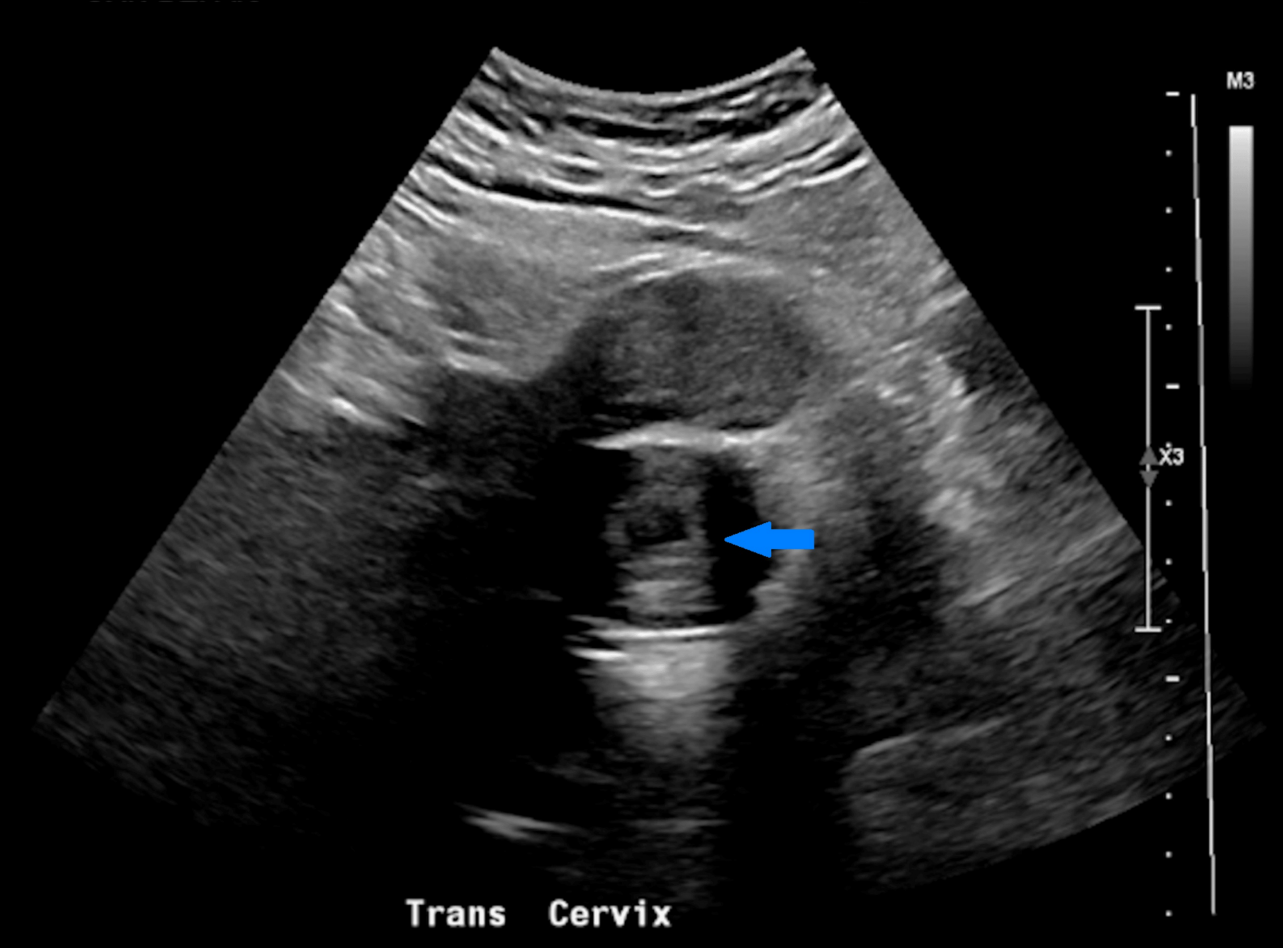
Transabdominal ultrasound on Day 0 demonstrating cervical ectopic pregnancy. Transabdominal ultrasound performed on Day 0 demonstrates a gestational sac implanted within the cervical canal (blue arrow), below the level of the internal os, consistent with a cervical ectopic pregnancy.

Day 0 (Initial Presentation): The patient presented with vaginal bleeding at approximately 6 weeks of gestation. Transvaginal ultrasound demonstrated a gestational sac implanted within the cervical canal with CRL 0.3-0.4 cm (Figures 1-2). Cardiac activity had been documented transiently earlier in the week but was not visualized at this visit. Mean sac diameter was consistent with 6-week gestational age. β-hCG measured 14,632 mIU/mL (reference value <5 mIU/mL). Hemoglobin was 14.6 g/dL (reference range 12.0-16.0 g/dL), and she was hemodynamically stable.

Given her desire for fertility preservation, absence of contraindications, and stability, she elected multi-dose systemic MTX with oral leucovorin 10 mg every 2 days (Table 1).

**Table 1:** Timeline of clinical course, β-hCG, interventions, and key findings/outcomes. β-hCG: beta human chorionic gonadotropin; MTX: methotrexate; TVUS: transvaginal ultrasound; UAE: uterine artery embolization; D&C: dilation and curettage; EBL: estimated blood loss

Day (Relative to Presentation)	β-hCG (mIU/mL) - (Reference Value <5 mIU/mL)	Intervention(s)	Key Findings/Outcomes
Day 0	14,632	MTX dose #1 (50 mg/m² IM)	TVUS: gestational sac in cervix, CRL ~3 mm, no cardiac activity
Day 1		Oral leucovorin 10 mg	
Day 2	12,717	MTX dose #2 (1 mg/kg IM)	13% decline in β-hCG
Day 3		Oral leucovorin 10 mg	
Day 4	9,555	MTX dose #3 (1 mg/kg IM)	25% decline in β-hCG
Day 5		Oral leucovorin 10 mg	
Day 6	9,695	MTX dose #4 (1 mg/kg IM)	Plateau; <2% change, consistent with MTX failure
Day 7		Oral leucovorin 10 mg	
Day 8	9,008	—	7% decline in β-hCG; persistent bleeding
Day 13	—	Bilateral UAE + suction D&C	EBL 5 mL; products evacuated; pathology confirmed gestational tissue
Follow-up	Declining to negative (trend ongoing)	—	Patient stable; fertility preservation counseling provided

Throughout this period, the patient experienced persistent light vaginal bleeding without hemodynamic instability. Laboratory studies revealed mild transaminitis (AST 39 U/L (reference range 10-40 U/L); ALT 86 U/L (reference range 7-56 U/L)), but values remained <3× the upper limit of normal and were not considered a contraindication to therapy.

By Day 6, with less than 15% decline in β-hCG after the 4th dose of methotrexate, medical treatment was considered unsuccessful per established thresholds [11].

Day 13 (Definitive Management): After multidisciplinary counseling, in the setting of plateauing β-hCG levels following multiple doses of methotrexate, the patient elected for surgical management with fertility preservation. Bilateral UAE with absorbable gelatin powder was performed, followed immediately by ultrasound-guided suction D&C. Intraoperative findings included a gestational sac protruding from the external os. Products of conception were evacuated intact with a suction curette under ultrasound guidance. A small cervical laceration occurred, controlled with an Allis clamp and Monsel’s solution.

Estimated blood loss was 5 mL. No transfusion was required. Pathology confirmed gestational tissue.

The patient recovered uneventfully and was discharged the same day. At postoperative counseling, she was advised to delay conception attempts for 3-6 months to allow MTX washout and vascular recovery. 

## Discussion

CEP remains a rare clinical entity in both natural and assisted conceptions. In the general population, cervical ectopic pregnancies account for less than 1% of all ectopic pregnancies, with an overall incidence estimated at less than 0.01% of all pregnancies [1,2]. By contrast, the incidence of ectopic pregnancy overall in the general population is approximately 1-2% of all pregnancies [1,3,4]. In the context of ART, large-scale data indicate that the incidence of CEP is 0.035% (3.5 per 10,000) of all ongoing ART pregnancies, and cervical ectopic pregnancies comprise 2.02% of all ectopic pregnancies following ART [5]. These findings suggest that, while ART is associated with an increased risk of ectopic pregnancy overall, cervical implantation remains an uncommon site even in this higher-risk population [2,4,5].

The observed differences in incidence between ART and non-ART populations likely reflect a combination of underlying patient risk factors, procedural variables inherent to ART, and improved detection and reporting [1,4,5]. In our patient, several elements of her reproductive history and pre-IVF evaluation probably contributed to the development of CEP. She had surgically confirmed bilateral tubal occlusion and a left hydrosalpinx in the setting of extensive pelvic adhesions and endometriosis. Tubal disease and hydrosalpinx are associated with abnormal implantation and reduced implantation and live-birth rates [1,4]. Her pelvic adhesive disease further distorted pelvic anatomy and may have impacted embryo migration and endometrial receptivity. In addition, cervical stenosis requiring dilation prior to embryo transfer created technical challenges. Difficult transfers, deep catheter placement, use of a tenaculum, and repeated manipulations are recognized risk factors for cervical implantation in ART cycles [2,5]. Thus, her baseline infertility factors and transfer characteristics converged to increase her risk for CEP.

Early and accurate diagnosis is critical because delayed recognition can lead to catastrophic hemorrhage and loss of fertility. In this case, diagnosis was established by a constellation of classic sonographic findings: a gestational sac located entirely within the cervical canal, absence of an intrauterine pregnancy, a negative sliding sign, and peritrophoblastic vascularity on Doppler interrogation. These findings align with the sonographic diagnostic framework originally proposed by Jurkovic et al. to distinguish CEP from low intrauterine gestations or incomplete abortions [3,6,13].

Systemic or local MTX is often considered first-line therapy in hemodynamically stable patients; however, numerous studies have identified prognostic factors that strongly predict treatment failure. Hung et al. identified β-hCG ≥10,000 mIU/mL, gestational age ≥9 weeks, CRL >10 mm, and embryonic cardiac activity as the strongest predictors of MTX resistance in cervical pregnancy [11]. Reduced efficacy of MTX in viable cervical pregnancies has also been reported [12,14]. Our patient exhibited several unfavorable features: an initial β-hCG exceeding 14,000 mIU/mL, documented early embryonic cardiac activity, and gestational age near six weeks. Although her CRL was <10 mm, the cumulative risk profile was high [11,12,14]. As anticipated, her β-hCG plateaued despite multiple systemic MTX doses, with less than a 15% decline by Day 6, consistent with medical treatment failure according to established thresholds [11]. This underscores the importance of early risk stratification when counseling patients about medical therapy and setting expectations for potential transition to surgical management.

Historically, surgical evacuation of CEP, particularly with D&C alone, was associated with devastating complications, including massive hemorrhage and hysterectomy in a substantial proportion of cases [6-8]. The cervix’s rich vascularity and poor contractility, compared with the uterine corpus, contribute to the heightened risk of uncontrolled bleeding. Multiple case reports document life-threatening hemorrhage during or after D&C for cervical pregnancy, requiring emergent UAE, balloon tamponade, cerclage, or hysterectomy to achieve hemostasis [2,6-10]. In a scoping review of 454 cases, Fowler et al. reported that 9% of patients ultimately required hysterectomy and that more than half needed multiple interventions to achieve resolution, highlighting the complexity and potential morbidity of treating this condition [7].

Over the past two decades, management strategies have shifted toward multimodal, fertility-sparing approaches that incorporate hemostatic adjuncts to reduce hemorrhagic risk. UAE performed immediately before D&C has been shown in multiple series to minimize intraoperative blood loss and enable safe evacuation of cervical gestations [15,19]. Fylstra reported successful management of 13 consecutive CEP cases using ultrasound-guided suction curettage combined with balloon tamponade, achieving excellent hemostatic control and preserving fertility [16]. De La Vega et al. described a protocol combining cervical cerclage, intracervical carboprost, suction curettage, and balloon tamponade, which effectively prevented severe hemorrhage [9], and Pereira et al. reported control of acute hemorrhage in residual cervical pregnancy using curettage, tamponade, and cerclage [10]. Local injection of diluted vasopressin into the cervix surrounding the gestational sac can induce vasoconstriction and smooth muscle contraction, facilitating curettage with minimal blood loss [17]. Additional combined medical-interventional approaches, such as those described by Murji et al. and Mininni et al., further support the use of multimodal, fertility-preserving strategies [14,18]. Collectively, these reports support the concept that D&C, once considered prohibitively risky in CEP, can be performed safely when paired with prophylactic hemostatic measures and careful multidisciplinary planning [7-11,14-19].

Our management pathway, bilateral UAE followed immediately by ultrasound-guided suction curettage, was selected to minimize hemorrhagic risk while preserving reproductive potential. This strategy is supported by case series demonstrating that UAE, prior to curettage, significantly reduces blood loss, transfusion requirements, and the need for emergent hysterectomy [7,11,15,16,19]. In our patient, estimated blood loss was only 5 mL; no transfusion was required, and a small cervical laceration was easily controlled with conservative measures. Postoperatively, her β-hCG levels declined steadily to the non-pregnant range on serial outpatient testing, and she experienced an uncomplicated recovery without delayed hemorrhage or evidence of retained gestational tissue. She was counseled to delay conception attempts for 3-6 months to allow MTX washout and vascular recovery, and she remains an appropriate candidate for future embryo transfer.

Beyond acute management, reproductive prognosis after CEP is a critical consideration, especially for patients who conceived via ART. Historically, hysterectomy was frequently required to control hemorrhage, effectively eliminating fertility potential [6,7]. With the adoption of fertility-preserving strategies such as systemic or local MTX, UAE, balloon tamponade, cervical cerclage, and conservative surgical approaches, subsequent reproductive outcomes have improved substantially [9-11,14-16,18,19]. Fylstra noted resumption of regular menses in nearly all conservatively managed cases, with spontaneous or ART-assisted intrauterine pregnancies subsequently achieved by many patients [16]. Hu et al. reported a series of 19 patients treated with UAE followed by curettage, all of whom resumed normal menstruation, with several achieving subsequent intrauterine pregnancies [19]. More recent reports by Murji et al. and Mininni et al. further support combined medical-interventional strategies that preserve uterine integrity while achieving definitive treatment of CEP [14,18]. Our case adds to this growing body of evidence, demonstrating that UAE, followed by ultrasound-guided suction curettage, can provide definitive, fertility-sparing treatment in the setting of MTX-resistant CEP.

In summary, this case demonstrates the limitations of MTX therapy in a high-risk CEP following ART, highlights the role of UAE in reducing hemorrhagic complications, and illustrates how pre-existing infertility factors, including hydrosalpinx, endometriosis, cervical stenosis, and technically difficult embryo transfer, may predispose to abnormal implantation. Clinicians should maintain a high index of suspicion for CEP in patients with these risk factors, anticipate MTX resistance in those with β-hCG levels >10,000 mIU/mL, embryonic cardiac activity, or advancing gestational age, and be prepared to transition early to surgical strategies incorporating hemostatic adjuncts. In women pursuing fertility, particularly those undergoing ART, UAE followed by conservative curettage represents a safe and effective treatment pathway that balances maternal safety with reproductive potential [7,9-11,14-16,18,19].

## Conclusions

This case illustrates successful fertility-preserving management of CEP after FET complicated by methotrexate failure in a high-risk clinical setting. Despite an unfavorable profile for medical therapy, including elevated β-hCG and prior embryonic cardiac activity, careful monitoring allowed early recognition of treatment failure and timely transition to surgical management. Bilateral UAE followed immediately by ultrasound-guided suction D&C provided definitive treatment with minimal blood loss, no transfusion, and rapid postoperative β-hCG normalization, while avoiding hysterectomy.

For clinicians caring for patients who conceive with ART, this case underscores the importance of maintaining a high index of suspicion for CEP, closely tracking β-hCG trends during methotrexate therapy, and engaging a multidisciplinary team early when prognostic factors for medical failure are present. In appropriately selected, hemodynamically stable patients, UAE combined with conservative surgical evacuation offers a safe, effective approach that balances maternal safety with preservation of future reproductive potential.
